# The Challenges of Implementing Comprehensive Clinical Data Warehouses in Hospitals

**DOI:** 10.3390/ijerph19127379

**Published:** 2022-06-16

**Authors:** François Bocquet, Mario Campone, Marc Cuggia

**Affiliations:** 1 Data Factory & Analytics Department, Institut de Cancérologie de l’Ouest, F-44805 Nantes-Angers, France; 2 Law and Social Change Laboratory, CNRS UMR 6297, Nantes University, F-40000 Nantes, France; 3 Oncology Department, Institut de Cancérologie de l’Ouest, F-44805 Nantes-Angers, France; mario.campone@ico.unicancer.fr; 4 Center for Research in Cancerology and Immunology Nantes-Angers, INSERM UMR 1232, Nantes University and Angers University, F-44307 Nantes-Angers, France; 5 LTSI, INSERM UMR 1099, University of Rennes 1, LTSI, F-35042 Rennes, France; marc.cuggia@univ-rennes1.fr

Digital health, e-health, telemedicine—this abundance of terms illustrates the scientific and technical revolution at work, made possible by high-speed processing of health data, artificial intelligence (AI), and the profound upheavals currently taking place and yet to come in health systems. Far from being limited to facilitating the transmission of data and constituting an aid to medico-technical or medico-administrative management in hospitals, Big Data opens up unprecedented prospects for monitoring the state of health of populations, decision support in medicine, or risk characterization [1]. It is now accepted that the AI and Big Data and revolution will profoundly transform medical practices, care, and health research. Their applications in medicine are numerous, from fundamental research to diagnostic, predictive or therapeutic decision support tools [2,3,4]. The development of data science and bioinformatics is therefore essential for modeling the impact of multiple anomalies on the organism at the individual level. In the short term, monitoring a single patient could thus lead to the generation of several billion data points that would be made exploitable by Big Data to deal with the complexity of diseases and take all their dimensions into account [5]. Aggregating all this data will thus make it possible to model diseases during their various stages of development and help understand them. Simulation and modeling tools will make it possible to validate predictive algorithms for the evolution of a disease, optimize treatments, improve their effectiveness, or reduce their toxicity [6,7]. To learn, function and improve, AI, algorithms and models need to be fed with massive quantities of data that are as structured as possible and of high quality. Hospitals, whether public or private, have a major role to play in this transformation because they concentrate the richest data that is closest to clinical reality. It is in this context, and to respond to the challenge of reusing data generated by patient care, that health data warehouses are emerging for 10 years in hospitals. While the large-scale use of these data can lead to significant technological and medical progress, it also raises many questions regarding their: 1. heterogeneity, structuration, interoperability; 2. temporality, purpose of use; 3. quality and storage. In addition to these questions, there is the issue of the legal and ethical framework for reusing these data (4.).

To advance our knowledge, improve care and speed up research, it is urgent that this data pool be made usable. However, most of the time, hospital data are very heterogeneous, unstructured, and produced at different scales.

**1.**  **The Issues of Heterogeneity, Structure, and Interoperability of Hospital Health Data**

Data science can help us structure and standardize hospital data, but it is not enough. This work necessarily also involves considerable mobilization of the health professionals who “produce” the data. Let us make no mistake, beyond the large volume of incredibly diverse data accumulated at high speed in hospitals, the full potential of this mass of information is conditioned by the capacity of the data producers to analyze it and then draw reliable results from it. This question refers to two attributes of health data: its structuring (is it structured or not? Does this data benefit from a standardized structure based on a nomenclature or not?) and its quality (does the data meet quality standards enabling us to say that it is interpretable and complete?). It is impossible to be satisfied with erroneous or fragmentary clinical data or poor-quality medical imaging, which would only lead the AI algorithms to results that are not very robust or to false modeling. It is to meet this dual requirement of data structuring and quality that more and more hospitals have decided to develop their own comprehensive clinical data warehouses [8,9]—or to be part of national, regional, or subregional networks with a DWH—containing all the information resulting from the care of their patients [10,11].

Electronic Health Records (EHR) are increasingly used for real-world evidence studies—i.e., studies carried out based on data collected in current care practice outside the traditional framework of clinical trials—which require accurate data to assess medical or therapeutics outcomes [12,13]. Prior to this exploitation, several problems must be resolved, including technical ones concerning the structuring and quality of the source data, their interoperability, and their integration into these DWH. Patients are cared in hospitals by multidisciplinary teams over sometimes long periods and generate huge volumes of data. Due to the wide variety of data sources and the different environments in which they are produced, health data are by nature extremely heterogeneous in terms of typology and format. The variety in the data also comes from the fact that, for the same data source, the data can be in very different formats. For example, the textual data in a medical report may be in different formats or describe the same thing in different ways. Broadly speaking, a distinction can be made between ‘unstructured’, ‘semi-structured’ and ‘structured’ data. The first type—by far the most widespread, as it represents 80% of computerized patient data in health care institutions [14]—refers, for example, to textual data such as those found in hospitalization, consultation, anatomopathology, and multidisciplinary consultation meetings reports. Natural language processing (NLP) algorithms can be used to analyze unstructured documents with high speed and accuracy. Another example of unstructured data are medical images. It should be noted that these unstructured imaging data may nevertheless be accompanied by metadata making it possible to understand the context in which the data is created. In the case of images, the DICOM (Digital imaging and communications in medicine) standard is intended to play this role [15]. “Semi-structured” or partially structured data is an intermediate type of data between unstructured and structured data. These data can be described by attributes that can facilitate their structuring. Technically, it is data represented in a tag-based computer language such as XML (eXtensible Markup Language). Medical questionnaires or any other document stored in the Clinical Document Architecture (CDA) format of the HL7 (Health Level 7) standard are examples of semi-structured data [16]. Finally, data is said to be “structured” when it is formatted and transformed into a well-defined data model. Structured data are described with a repository allowing them to be enriched with semantics and thus making their exploitation or analysis possible. This description can be standard and then either local or shared by several data producers, which complicates the interoperability of the systems producing them [17].

**2.**  **Temporality and Purpose of the Hospital Data: Two Key Points**

An inherent element of data is its temporality. Repeated collection of data can allow them to be represented in the form of chronological series or sequences. This is the case for example for physiological measurements that can be performed on patients. Conventional biological analysis data, for example, have a temporality that may be useful to analyze to assess the evolution of biological parameters. These data are then called signal data in the sense that they can be defined by their acquisition frequency. The notion of temporality can also embrace a wider domain, for example in the context of reconstructing healthcare pathways. This exercise most often involves the use of unstructured data and is not always simple in practice [18,19].

The purpose for which the data are produced also has an impact on their characteristics and therefore on their quality. On this point, it is possible to note that, for the same information, the level of requirement in terms of quality is not the same in the case of a clinical trial or in routine care. While the experimental scheme of the clinical trial provides for the collection of data within a normalized, standardized framework, the data filled in by health professionals in EHR corresponding to their routine care—so-called “real-life” data—are often partial or incomplete [12]. Generally, as soon as a data source is created for study purposes, the data is structured: data from clinical studies, disease registers, or even Diagnosis Related Group (DRG) data in the medico-administrative field used for the reimbursement of care by health insurance. It should be noted that DRG data often do not have the same needs in terms of medical description of patients as care. In a logic of reuse of these data, it is essential to consider this, as these data describing the same medical information in different ways will potentially have to be reconciled [18].

**3.**  **The Central Issue of Data Quality and Storage in Hospitals**

The quality of the data is primarily a function of the purpose for which it is used and the structural, normalization and standardization requirements of its use. This approach is at the heart of the principle of the “fitness for use” approach [20]. Data quality can be assessed by taking the different characteristics of the data into account. Evaluating the intrinsic quality of a data item involves seeking a compromise on the level of quality of each of these components in order to meet a predefined study objective [18,21]. In terms of data quality, certain elements are commonly analyzed: missing data, duplicate data, the time required to produce the data, or the invalidity of the data. It should be remembered that from the perspective of secondary data re-use, uses are defined after the data have been produced. Beyond the characteristics to be determined by the subsequent use of the data, they can nevertheless be judged as being of ‘sufficient’ quality if they meet a minimum of criteria described by the ‘FAIR’ principles (Foundable, Accessible, Interoperable, Reusable) [22]. There are several ways in which data quality can be improved to enable reuse downstream of data production: developing quality monitoring measures throughout the data integration process to ensure that raw data is not degraded during the integration process from sources; developing analysis methods to correct data quality issues (reconciliation, deduplication, etc.). It is also possible to intervene upstream by applying corrective actions on the source applications, which is sometimes facilitated by the fact that the end users are also the data producers. Secondary reuse involves defining the dimensions of interest in terms of data quality in relation to the intended uses to implement indicators for assessing and monitoring data quality [18]. If massive health data can be described through the classic definition of the “5 V’s” of big data (volumetry, variability, veracity, velocity, and value), it is also possible to define them by the technological means necessary to exploit them. The traditional means of storage (relational databases) and calculation are no longer sufficient and recourse to other storage and calculation technologies (distributed calculations, supercomputers, etc.) is required [23]. As for the criteria of veracity, velocity, or value, these are likely to concern any type of data and are very dependent on the intended use. As regards variability or volumetry, they apply differently depending on the type of data concerned. For example, digitized medical imaging data or omics data meet the criterion of volumetry, but less often that of variability. Conversely, the electronic data traditionally contained in a patient file, while highly variable, represent only a limited volume at the scale of an institution. In all cases, storage and analysis methods must be adapted to take into account the massive nature of the data to be used [18].

**4.**  **Regulatory and Ethical Requirements for Hospital DWH**

While the exploitation of large amounts of health data is a source of progress and medical innovation, it legitimately raises questions of a legal and ethical nature. As with all data warehouses, because of the sensitivity of the data processed and for ethical reasons, the use of hospital data warehouses must be subject to strict rules on the processing of patients’ personal data. However, most often, several legal and ethical issues are still under debate: patients’ rights regarding the modalities of implementation of the DWH; solidarity and data as a common good; transparency and trust; and protection of individuals regarding the processing of personal data. As an example, this is the case in Europe [24]. Until recently, the European ethical-legal frameworks in force were not adapted to these DWHs because they were not conceived for re-using data in a different context than the one in which they were acquired. For that matter, access modalities to DWH must ensure the respect of patients’ rights: information to the patient, as well as confidentiality and security. As in other countries around the world, secondary use of the data is confronted with conflicting requirements with, on the one hand, the principle of open science (transparency and data sharing), the possibilities offered by Big Data and the reuse of healthcare or research data, and on the other, changes to the regulatory and legislative framework—including the general data protection regulation (GDPR) in the EU—and some additional national legislation [25]. As the complexity of the data flow increases, greater transparency and standardization of criteria and procedures are required to maintain objective oversight and control. The development of practice-oriented and evidence-based policies in this field is crucial [26,27]. On this point, it is interesting to note the recent initiative of the National Commission for Data Protection and Liberties in France (CNIL). This commission has the task of assisting professionals in complying with their obligations and helps individuals to control their personal data and exercise their rights. The CNIL drew up a standard dedicated to the hospital DWH at the end of 2021 in order to specify the legal framework, resulting from the General Data Protection Regulation (GDPR) and national provisions, applicable to them. The following fields are covered by the standard: governance, nature of the data they contain, purposes of data processing and arrangements for access to data, obligations to inform patients about the collection and use of their data, arrangements for exercising patients’ rights of access and opposition, rules on storage, etc. [28].

All over the world, researchers and clinicians face major obstacles using hospital data because of a lack of international standards regarding data characterization and quality. Despite these barriers, the number of data-sharing initiatives continues to grow. The central issue is to progressively evolve towards a multi-domain and multi-scale integration of health data, which is the only way to reconstitute dimensions ranging from the genome to the exposome. Regarding the structuring of data, it is not possible today to structure everything to start working (ETL process extracting from application sources, transforming to load into a schema). In the world of Big Data things are changing to a more agile approach where we do ELT (extract from sources, load and transform as needed with the logic of data lakes). The structuring of data upstream at the application level is essential but by no means sufficient and suitable for carrying out studies on real life data. Regarding the structuring of data, it is necessary to be more vigilant about everything that guarantees it throughout the data production chain, from the patient’s bed to the integration. If DWHs constitute a de-siloing and a provision of data so that the hospital can regain control of its data, it is essential to underline that DWHs will not be able to solve everything and that it is still humans who will be at the helm, which is rather reassuring. The aim of this Special Issue is to address all these questions and to make substantial contributions to knowledge gaps in understanding the scientific and methodological issues related to structuring and qualifying the data that feed hospital data warehouses and their potential impact on research and public health.
